# Health, ageing and private health insurance: baseline results from the 45 and Up Study cohort

**DOI:** 10.1186/1743-8462-6-16

**Published:** 2009-07-13

**Authors:** Emily Banks, Louisa Jorm, Sanja Lujic, Kris Rogers

**Affiliations:** 1 National Centre for Epidemiology and Population Health, Australian National University, ACT 0200, Australia; 2 The Sax Institute, PO Box 123, Broadway NSW 2007, Australia; 3 School of Medicine, University of Western Sydney, Campbelltown Campus, Locked Bag 1797, Penrith South DC NSW 1797, Australia

## Abstract

**Background:**

This study investigates the relationships between health and lifestyle factors, age and private health insurance (PHI) in a large Australian population-based cohort study of people aged 45 years and over; the 45 and Up Study. Unlike previous Australian analyses of relationships between health, lifestyle and PHI, it incorporates adjustment for multiple confounding socioeconomic and demographic factors. Recruitment into the 45 and Up Study began in February 2006 and these analyses relate to the first 103,042 participants who joined the study prior to July 2008.

**Results:**

The proportion with PHI decreased with increasing age. The factors independently and most strongly associated with having PHI were: higher income; higher educational attainment; not holding a health care concession card; not being of Aboriginal/Torres Strait Islander origin; being a non-smoker; high levels of self-rated health and functional capacity; and low levels of psychological distress. These factors increased the probability of having PHI by 16% to 125%, compared to individuals without these characteristics. PHI coverage was significantly but only marginally higher in people reporting non-melanoma skin cancer (adjusted RR 1.04, 95%CI 1.03–1.05), prostate cancer (1.09, 1.06–1.11) or an enlarged prostate (1.07, 1.06–1.09), those reporting a family history of a range of conditions (e.g. 1.02, 1.01–1.03 for a family history of heart disease; 1.03, 1.02–1.04 for a family history of prostate cancer) and lower in people reporting diabetes (0.92, 0.91–0.94) or stroke (0.91, 0.88–0.94), compared to people who did not have these medical or family histories. PHI was higher in those reporting certain surgical procedures with RRs (95%CI) of 1.12 (1.09–1.15) for hip replacement, 1.10 (1.08–1.13) for knee replacement and 1.12 (1.09–1.15) for prostatectomy, compared to those not reporting these interventions.

**Conclusion:**

Compared to the rest of the study population, those with PHI are richer, better educated, more health conscious, in better health and more likely to use certain discretionary health services. Hence, PHI use is generally highest among those with the least need for health care. Whether or not people have PHI is more strongly associated with demographic and lifestyle factors than with health status.

## Background

The Australian health care system incorporates a complex mixture of public and private sector involvement. While universal health care is provided by state and federal governments, tax and other incentives encourage individuals to take out private health insurance (PHI), particularly to cover hospital costs.

The introduction of Medicare, Australia's universal health care scheme, in 1984 resulted in a steady decline in the proportion of the Australian population covered by PHI [1]. The Australian Government's 30% tax rebate for private health insurance premiums began in 1999, and the Lifetime Health Cover policy began in 2000 (whereby people taking up PHI after July 2000 pay a 2% premium loading for each year that their entry age is over 30). Population PHI coverage rates rose rapidly from 31% in June 1999 to 46% in September 2000 [1]. By June 2007, over nine million Australians had private hospital insurance cover (44% of the population) [2]. Recent changes to incentives have specifically targeted older people. From April 2005, the private health insurance rebate increased from 30% to 35% for persons aged 65–69 years and to 40% for persons aged 70 years and over [3].

An in-depth understanding of patterns of PHI among the older population is important not only for quantifying current and ongoing costs of healthcare in Australia, but also because those with PHI tend to have better access to certain elements of health care, especially elective surgery. Little is known about how demographic, health and lifestyle factors relate to PHI status. This study investigates the relationships between health and lifestyle factors, age and PHI in a large Australian population-based cohort study of people aged 45 years and over; the 45 and Up Study. Unlike previous Australian analyses of relationships between health, lifestyle and PHI, our study incorporates adjustment for multiple confounding socioeconomic and demographic factors. This is made possible by the detailed information collected in the Study's baseline questionnaire, and its inclusion of very large numbers of participants from across age and socioeconomic strata.

## Methods

### Study population

The 45 and Up Study is described in detail elsewhere [4]. Briefly, the 45 and Up Study is a large scale study of healthy ageing that involves 250,000 men and women aged 45 years and over from the general population of New South Wales, the most populous state in Australia. Individuals aged 45 years and over are sampled from the Medicare Australia database, which provides virtually complete coverage of the general population, and join the study by completing a postal questionnaire and providing written consent to follow their health in the long term, through repeat questionnaires, linkage to health records and additional more detailed data collection. There is two-fold oversampling of individuals aged 80 and over and those resident in rural areas. For the first 34,645 study participants those with an active Medicare card were sampled, i.e. those who had had some use of government funded health services in the previous 6 years. Subsequent to this, eligibility was changed slightly to include those who had had used a health service in the previous 2 years, to reduce mailings to deceased individuals. Data from Medicare Australia indicate that 97% of the NSW general population aged 45 and over meet this criterion.

Recruitment into the study began in February 2006 and these analyses use information from the 103,042 participants who joined the study before July 2008.

### Definitions, classification and exclusions

All of the variables used in this study were derived from self-reported data from the 45 and Up Study questionnaire (available at ), apart from the measure of remoteness of residence, which was assigned according to the mean Accessibility Remoteness Index of Australia Plus (ARIA+) [5] score for the postcode of the participant's residential address as recorded by Medicare. Variables were classified according to the groupings in Table 1.

**Table 1:** Sociodemographic factors and private health insurance status

	**Private health insurance**				
**Demographics**	N	% column	% PHI	RR crude*	RR adjusted^†^
**Sex**					
Male^‡^	45,958	47.0	65.4	1	1
Female	51,881	53.0	64.9	0.99 (0.98, 1.00)	1.06 (1.05, 1.07)
**Age**					
45 – 49^‡^	12,372	12.6	66.7	1	1
50 – 54	16,083	16.4	69.0	1.03 (1.02, 1.05)	1.06 (1.04, 1.07)
55 – 59	17,172	17.6	70.0	1.05 (1.03, 1.07)	1.14 (1.12, 1.16)
60 – 64	15,100	15.4	68.7	1.03 (1.01, 1.05)	1.22 (1.20, 1.24)
65 – 69	12,539	12.8	63.8	0.96 (0.94, 0.97)	1.25 (1.23, 1.27)
70 – 74	9,133	9.3	57.8	0.87 (0.85, 0.88)	1.23 (1.21, 1.25)
75 – 79	6,726	6.9	56.7	0.85 (0.83, 0.87)	1.26 (1.23, 1.28)
80 – 84	6,116	6.3	57.4	0.86 (0.84, 0.88)	1.27 (1.24, 1.30)
85+	2,598	2.7	53.3	0.80 (0.77, 0.83)	1.29 (1.24, 1.33)
**Card possession**					
No^‡^	69,337	70.9	78.0	1	1
Yes – Health care card	28,502	29.1	33.8	0.43 (0.43, 0.44)	0.52 (0.51, 1.53)
**Remoteness (ARIA+)**					
Major city^‡^	42,279	43.2	71.7	1	1
Inner regional	35,317	36.1	62.5	0.87 (0.86, 0.88)	0.90 (0.89, 0.91)
Outer regional	18,121	18.5	55.8	0.78 (0.77, 0.79)	0.83 (0.82, 0.84)
Remote/very remote	2,001	2.0	58.5	0.82 (0.79, 0.85)	0.90 (0.87, 0.93)
**Pre-tax household income (AUD)**					
0 to <20,000^‡^	19,314	19.7	34.9	1	1
20,000 to 49,999	24,566	25.1	63.6	1.82 (1.78, 1.86)	1.69 (1.66, 1.73)
50,000 to 69,999	10,262	10.5	77.3	2.22 (2.17, 2.26)	2.02 (1.98, 2.07)
≥ 70,000	22,112	22.6	90.0	2.58 (2.53, 2.63)	2.25 (2.20, 2.30)
Did not disclose	16,469	16.8	66.7	1.91 (1.87, 1.95)	1.78 (1.74, 1.82)
**Highest educational qualification**					
None^‡^	11,569	11.8	38.8	1	1
Trade	10,833	11.1	55.8	1.44 (1.40, 1.48)	1.31 (1.27, 1.34)
School certificate	21,364	21.8	59.9	1.55 (1.51, 1.59)	1.38 (1.34, 1.41)
HSC	9,375	9.6	66.0	1.70 (1.66, 1.75)	1.48 (1.44, 1.52)
Certificate/diploma	20,343	20.8	71.6	1.85 (1.80, 1.89)	1.53 (1.49, 1.57)
University	22,648	23.1	83.0	2.14 (2.09, 2.19)	1.62 (1.58, 1.66)
**Aboriginality**					
Non Aboriginal^‡^	95,124	97.2	65.7	1	1
Aboriginal/TSI	803	0.8	34.4	0.52 (0.48, 0.58)	0.63 (0.58, 0.68)
**Relationship status**					
No partner^‡^	23,297	23.8	50.0	1	1
Partner	74,290	75.9	69.9	1.40 (1.38, 1.42)	1.21 (1.19, 1.22)
**Work status**					
In paid work^‡^	46,123	47.1	74.3	1	1
Retired	40,628	41.5	59.8	0.80 (0.80, 0.81)	1.21 (1.19, 1.23)
Other	10,387	10.6	46.8	0.63 (0.62, 0.64)	1.06 (0.96, 1.18)
**Country of birth**					
Australia^‡^	72,790	74.4	67.3	1	1
Other	24,121	24.7	59.1	0.88 (0.87, 0.89)	0.86 (0.85, 0.86)

^† ^Adjusted, where appropriate, for age, sex, remoteness, income, education, aborginality, relationship status and country of birth

^‡ ^Reference category

Percentages do not consistently total to 100% due to missing values

Regarding health insurance status, participants were asked "Which of the following do you currently hold?" and were given a range of options relating to private health insurance and government and war veterans' health benefit cards. Participants were classified as having "hospital cover PHI", if they indicated they had "PHI without extras", which essentially covers hospital and specialist care only, and as having "combined hospital and ancillary PHI" if they indicated that they had "PHI with extras", which additionally covers services such as dental care, allied health and optometry. These two groups were combined to form a general category of those holding PHI.

Psychological distress was measured using the Kessler-10 score [6] and functional capacity using the Medical Outcomes Study Physical Functioning scale; a lower physical functioning score indicates more severe functional limitation [7]. Kessler-10 scores were classified into 4 groups: low psychological distress (score 10–15), moderate psychological distress (score 16–21), high psychological distress (score 22–29) and very high psychological distress (score 30 or higher). Functional limitation scores were classified into 5 groups: no limitation (score of 100), minor limitation (score 95–99), mild limitation (score 85–94), moderate limitation (60–84) and severe limitation (score 0–59). Medical conditions were classified according to the response to the question "Has a doctor ever told you you have any of the following..." and operations in response to the question "Have you ever had any of the following operations..."

A small number of participants (n = 3,124) who reported holding a gold card from the Department of Veterans' Affairs were excluded, since this entitles them to a very wide range of services and means they were not strictly comparable to the rest of the study population in terms of their eligibility to use PHI. A further 2,079 participants who were missing data on PHI were also excluded.

### Statistical methods

Differences between those with any PHI versus no PHI were tested using the chi-square test. Relative risks (RR) and 95% CIs were estimated by generalized linear models, specifying Poisson distribution with a robust error variance [8]. Both crude and adjusted relative risks were computed; unless otherwise specified, descriptions refer to adjusted relative risks. Relative risks were adjusted for sex, age, remoteness, income, education, Indigenous status, relationship status and country of birth, using the categories in Table 1, with an additional category for missing values. Having a health care card was considered a potential mediating factor in the relationship between income and PHI and was therefore not adjusted for. Colinearity between being in paid work and income meant that adjustment for work status was not appropriate. For medical conditions, family history and operations the reference group is individuals who do not have the specified condition under investigation. For other variables of interest, the reference group is the lowest category in the list or the largest group.

All analyses were carried out in SAS V9 [9]. All statistical tests were two-sided, using a significance level of p < 0.01. Due to large sample size, conclusions were drawn based on both significance and the effect size.

## Results

Of a total of 97,839 participants, 63,742 (65.1%) reported having any PHI. 14,999 reported hospital cover PHI and 48,743 reported holding combined hospital and ancillary PHI.

Overall, 65% of men and women had PHI (Table 1). The crude proportion of people with PHI fell from 69–70% of those aged 50–64 to 64% of those aged 65–69, 57–58% of those aged 70–84 and 53% of those aged 85 or older. The adjusted results indicate that for a given sex, income, education level, remoteness, aboriginality, relationship status and country of birth, older people in the study had a *higher *probability of having PHI than younger people. The large change in the RR with adjustment indicates that the relationship between age and PHI is influenced substantially by the demographic factors listed in Table 1.

**Figure 1:**
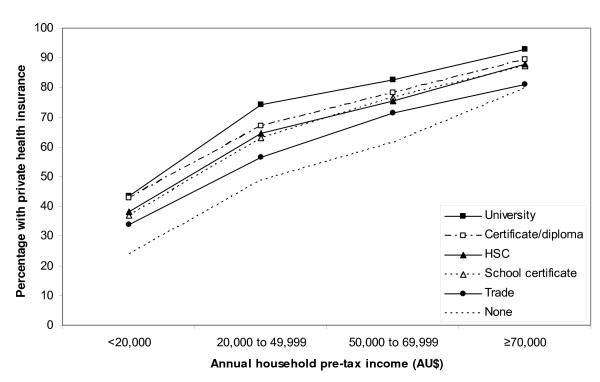
**Proportion of study participants with private health insurance by income and educational attainment**.

The adjusted RR of having any PHI was lower among those living outside major urban centres. Those with lower incomes, lower levels of education, a health care concession card and those reporting Indigenous origin, were significantly and substantially less likely to hold PHI than other members of the cohort (Table 1). The likelihood of holding PHI was significantly reduced, but to a lesser extent, in those who were not in a marriage-type relationship and those born outside of Australia. Figure 1 shows the proportion of individuals with any PHI according to both education and income and demonstrates the strong independent influence of both of these socio-economic factors on PHI uptake.

Compared to individuals of healthy weight, the adjusted RR of PHI was slightly elevated in those who were overweight and reduced in those who were obese (Table 2). Current and past smokers were significantly less likely to hold PHI than those who had never smoked. Those who reported drinking alcohol were more likely to hold PHI than those who did not drink weekly, although there was no trend with increasing consumption of alcohol. PHI was significantly more common in individuals who reported higher versus lower levels of physical activity and fruit and vegetable consumption and sufficient versus insufficient levels of these, according to current national guidelines [10,11].

**Table 2:** Health risk factors and private health insurance status, adjusted for sociodemographic factors

	**Private health insurance**				
**Lifestyle factors**	N	% column	% PHI	RR crude*	RR adjusted^+^
**BMI categories^‡^**					
Underweight	3,903	4.0	61.1	0.91 (0.89, 0.94)	0.97 (0.95, 0.99)
Healthy weight^§^	31,256	31.9	67.0	1	1
Overweight	36,027	36.8	67.4	1.01 (1.00, 1.02)	1.02 (1.01, 1.03)
Obese	19,619	20.1	61.0	0.91 (0.90, 0.92)	0.97 (0.96, 0.99)
**Smoking status**					
Never^§^	55,522	56.7	69.7	1	1
Past	34,989	35.8	62.8	0.90 (0.89, 0.91)	0.94 (0.93, 0.94)
Current	7,319	7.5	41.4	0.59 (0.58, 0.61)	0.71 (0.70, 0.73)
**Alcohol consumption (drinks/week)**					
Zero^§^	31,098	31.8	55.4	1	1
1 to 6	28,339	29.0	70.2	1.27 (1.25, 1.28)	1.11 (1.10, 1.12)
7 to 13	17,992	18.4	72.7	1.31 (1.29, 1.33)	1.12 (1.10, 1.13)
14 to 20	10,732	11.0	72.5	1.31 (1.29, 1.33)	1.11 (1.10, 1.13)
21 and over	7,642	7.8	63.0	1.14 (1.12, 1.16)	1.03 (1.01, 1.05)
**Physical activity (sessions/week)**					
Zero^§^	4,047	4.1	52.7	1	1
1 to 6	24,557	25.1	65.0	1.23 (1.20, 1.27)	1.09 (1.06, 1.12)
7 to 10	24,526	25.1	65.8	1.25 (1.21, 1.29)	1.08 (1.05, 1.11)
11 to 15	20,030	20.5	68.0	1.29 (1.25, 1.33)	1.10 (1.07, 1.13)
16 and over	22,810	23.3	65.8	1.25 (1.21, 1.29)	1.07 (1.04, 1.10)
**Vegetable intake (serves/day)**					
Zero^§^	1,994	2.0	47.8	1	1
>0 to 1	9,576	9.8	58.4	1.22 (1.16, 1.28)	1.08 (1.04, 1.13)
2 to 3	40,085	41.0	66.2	1.38 (1.32, 1.45)	1.14 (1.10, 1.19)
4 to 5	24,065	24.6	68.5	1.43 (1.37, 1.50)	1.17 (1.12, 1.22)
6 and over	20,035	20.5	65.0	1.36 (1.30, 1.42)	1.17 (1.12, 1.22)
**Fruit intake (serves/day)**					
Zero^§^	6,225	6.4	50.7	1	1
>0 to 1	19,018	19.4	62.5	1.23 (1.20, 1.26)	1.12 (1.09, 1.15)
2 to 3	53,961	55.2	67.4	1.33 (1.30, 1.36)	1.17 (1.15, 1.20)
4 and over	16,362	16.7	67.7	1.34 (1.30, 1.37)	1.19 (1.16, 1.22)

* Relative risk of having any private health insurance

^+ ^Adjusted for age, sex, remoteness, income, education, aborginality, relationship status and country of birth

^‡ ^BMI categories: Underweight (BMI<20), Normal weight (BMI 20-<25), Overweight (BMI 25-<30), Obese (BMI 30+)

^§ ^Reference category

Percentages do not consistently total to 100% due to missing values

The proportion of study participants reporting PHI decreased with increasing levels of psychological distress, with decreasing self-rated health and with reductions in functional capacity (Table 3). Although PHI was significantly elevated among people reporting a history of non-melanoma skin cancer, enlarged prostate and prostate cancer and was significantly reduced in those reporting stroke or diabetes, compared to those without these conditions, the observed variation was not large, with the most extreme RR being those for stroke (RR 0.91, 95% CI 0.88–0.94) and prostate cancer (RR 1.09, 95% CI 1.06–1.11). People reporting none of the illnesses listed on the questionnaire were significantly less likely to hold PHI than those reporting any illness. Lesser variations in the RR for PHI were observed according to family history of a range of specific diseases, with significantly but only slightly increased RRs of PHI in those with a family history of heart disease, hypertension, dementia, bowel cancer, osteoporosis, and prostate cancer and slightly reduced RR of PHI with a family history of diabetes and severe depression. In general, PHI was significantly more common among those with a history of a range of operations, including removal of skin cancer, knee and hip replacement, vasectomy, prostatectomy, hysterectomy and prolapse repair. PHI was significantly more common in those reporting previous screening for breast, prostate and colorectal cancer.

**Table 3:** Health status, health conditions, health actions and private health insurance status, adjusted for sociodemographic factors

	**Private health insurance**				
**Health conditions**	No.	% column	% PHI	RR crude*	RR adjusted^†^
**Psychological distress**					
Low^‡^	64,669	66.1	69.8	1	1
Moderate	13,223	13.5	62.8	0.90 (0.89, 0.91)	0.96 (0.95, 0.97)
High	4,515	4.6	51.7	0.74 (0.72, 0.76)	0.87 (0.85, 0.89)
Very high	2,736	2.8	47.1	0.68 (0.65, 0.70)	0.84 (0.81, 0.87)
**Overall health**					
Excellent^‡^	14,867	15.2	75.0	1	1
Very good	35,255	36.0	71.3	0.95 (0.94, 0.96)	1.01 (1.00, 1.02)
Good	31,598	32.3	62.4	0.83 (0.82, 0.84)	0.97 (0.96, 0.98)
Fair	10,875	11.1	48.5	0.65 (0.63, 0.66)	0.86 (0.85, 0.88)
Poor	1,898	1.9	35.5	0.47 (0.44, 0.50)	0.71 (0.67, 0.75)
**Functional limitation**					
No limitation	29,054	29.7	72.0	1	1
Minor limitation	14,903	15.2	73.6	1.02 (1.01, 1.03)	1.01 (1.00, 1.03)
Mild limitation	16,308	16.7	69.1	0.96 (0.95, 0.97)	1.00 (0.99, 1.01)
Moderate limitation	14,600	14.9	61.2	0.85 (0.84, 0.86)	0.97 (0.95, 0.98)
Severe limitation	13,146	13.4	47.2	0.66 (0.64, 0.67)	0.86 (0.84, 0.88)
**Number of conditions**					
None^‡^	21,553	22.0	66.7	1	1
1	22,727	23.2	67.8	1.02 (1.00, 1.03)	1.02 (1.01, 1.03)
2	21,311	21.8	64.8	0.97 (0.96, 0.98)	1.02 (1.00, 1.03)
3	10,023	10.2	61.8	0.93 (0.91, 0.94)	1.02 (1.00, 1.03)
4 or more	5,798	5.9	61.0	0.91 (0.89, 0.93)	1.04 (1.01, 1.06)
**Ever had^§^**					
Skin cancer	24,917	25.5	68.7	1.07 (1.06, 1.08)	1.04 (1.03, 1.05)
Melanoma	5,299	5.4	65.6	1.01 (0.99, 1.03)	1.02 (1.00, 1.03)
Other cancer	6,108	6.2	61.6	0.94 (0.92, 0.96)	0.99 (0.98, 1.01)
Heart disease	11,475	11.7	60.0	0.91 (0.90, 0.93)	0.99 (0.98, 1.01)
High blood pressure **	34,107	34.9	62.8	0.95 (0.94, 0.96)	1.00 (0.99, 1.01)
Stroke	3,102	3.2	50.2	0.76 (0.74, 0.79)	0.91 (0.88, 0.94)
Diabetes	8,526	8.7	53.9	0.81 (0.80, 0.83)	0.92 (0.91, 0.94)
Thrombosis	4,548	4.6	60.3	0.92 (0.90, 0.94)	1.00 (0.97, 1.02)
Parkinson's disease	672	0.7	58.5	0.90 (0.84, 0.96)	1.01 (0.96, 1.07)
None of the above	20,319	20.8	67.1	1.04 (1.03, 1.05)	0.98 (0.97, 0.99)
Breast cancer^¶^	2,675	5.2	64.7	1.00 (0.97, 1.03)	1.01 (0.99, 1.04)
Enlarged prostate^||^	7,576	16.5	67.8	1.04 (1.03, 1.06)	1.07 (1.06, 1.09)
Prostate cancer^||^	2,780	6.0	67.1	1.03 (1.00, 1.06)	1.09 (1.06, 1.11)
**Family history^§,††^**					
Heart disease	45,014	46.0	66.3	1.03 (1.02, 1.04)	1.02 (1.01, 1.03)
High blood pressure	48,909	50.0	67.1	1.06 (1.05, 1.07)	1.02 (1.01, 1.03)
Stroke	25,670	26.2	65.4	1.00 (0.99, 1.02)	1.00 (0.99, 1.01)
Diabetes	21,981	22.5	62.9	0.96 (0.95, 0.97)	0.98 (0.97, 0.99)
Dementia	15,877	16.2	68.3	1.06 (1.05, 1.07)	1.03 (1.02, 1.04)
Parkinson's disease	4,561	4.7	65.5	1.01 (0.98, 1.03)	0.99 (0.97, 1.01)
Severe depression	12,020	12.3	64.0	0.98 (0.97, 0.99)	0.97 (0.96, 0.98)
Severe arthritis	20,760	21.2	63.2	0.96 (0.95, 0.97)	1.00 (0.99, 1.01)
Breast cancer	10,991	11.2	66.0	1.02 (1.00, 1.03)	1.01 (0.99, 1.02)
Bowel cancer	13,471	13.8	66.5	1.02 (1.01, 1.04)	1.02 (1.01, 1.03)
Lung cancer	10,115	10.3	62.3	0.95 (0.94, 0.97)	0.98 (0.97, 1.00)
Melanoma	8,689	8.9	67.9	1.05 (1.03, 1.06)	1.00 (0.99, 1.02)
Osteoporosis	13,830	14.1	68.6	1.06 (1.05, 1.08)	1.03 (1.02, 1.04)
Hip fracture	9,720	9.9	66.8	1.03 (1.01, 1.04)	1.01 (1.00, 1.03)
Prostate cancer	9,960	10.2	68.5	1.06 (1.04, 1.07)	1.03 (1.02, 1.04)
Ovarian cancer	3,482	3.6	61.9	0.95 (0.92, 0.97)	0.99 (0.97, 1.02)
**Operations^§^**					
Removal of skin cancer	25,930	26.5	68.4	1.07 (1.06, 1.08)	1.04 (1.03, 1.05)
Knee replacement	3,829	3.9	64.2	0.99 (0.96, 1.01)	1.10 (1.08, 1.13)
Hip replacement	3,026	3.1	66.6	1.02 (1.00, 1.05)	1.12 (1.09, 1.15)
Gallbladder removed	9,803	10.0	60.5	0.92 (0.91, 0.94)	0.99 (0.97, 1.00)
Vasectomy^||^	10,998	23.9	71.9	1.13 (1.12, 1.15)	1.02 (1.01, 1.03)
Part of prostate removed^||^	2,661	5.8	67.3	1.03 (1.00, 1.06)	1.10 (1.07, 1.13)
Whole prostate removed^||^	1,653	3.6	71.6	1.10 (1.06, 1.13)	1.12 (1.09, 1.15)
Hysterectomy^¶^	14,827	28.6	62.9	0.96 (0.94, 0.97)	1.02 (1.01, 1.03)
Both ovaries removed^¶^	5,185	10.0	62.4	0.96 (0.94, 0.98)	1.03 (1.01, 1.05)
Sterilisation^¶^	14,311	27.6	64.1	0.98 (0.97, 1.00)	0.99 (0.98, 1.00)
Prolapse repair^¶^	5,916	11.4	63.9	0.98 (0.96, 1.00)	1.04 (1.03, 1.06)
**Screening history^§^**					
Bowel cancer screening	46,357	47.4	70.9	1.18 (1.17, 1.19)	1.12 (1.11, 1.13)
Breast cancer screening^¶^	45,708	88.1	66.5	1.24 (1.21, 1.27)	1.16 (1.13, 1.19)
Prostate disease screening^||^	31,549	68.6	69.1	1.20 (1.18, 1.22)	1.14 (1.12, 1.16)

* Relative risk of having any private health insurance

^† ^Adjusted for age, sex, remoteness, income, education, aborginality, relationship status and country of birth

^‡ ^Reference category ^§ ^Reference category = No

^||^Males only ^¶ ^Females only

** Excluding high blood pressure during pregnancy

^†† ^Whether mother, father, brother or sister had the disease

Percentages do not consistently total to 100% due to missing values

Similar patterns were seen when the data were split into hospital cover PHI and hospital and ancillary PHI (data not shown).

## Discussion

In this population-based study of Australians aged 45 and over, multiple factors were found to differ significantly between those with and without PHI. The factors independently and most strongly related to having PHI were: higher income; higher educational attainment; not holding a health care concession card; not being of Aboriginal/Torres Strait Islander origin; being a non-smoker; high levels of self-rated health and functional capacity; and low levels of psychological distress. Significant but lesser differences were observed according to other demographic factors, with higher levels of PHI in those who were younger, living in urban areas, those with a partner, those in paid work and those born in Australia, compared to other people. People who were of healthy weight, who drank alcohol and had sufficient levels of physical activity and fruit and vegetable intake, were more likely to have PHI. Finally, PHI coverage was higher in people reporting non-melanoma skin cancers, prostate cancer or an enlarged prostate, those reporting a family history of a range of conditions and those reporting surgery for a range of reasons, and lower in people reporting diabetes or stroke, compared to people who did not have these medical, surgical or family histories.

In this study, older people were less likely than those aged under 60 to have PHI, in absolute terms. However, the finding of a large change in the RR of having PHI following adjustment for demographic variables indicates that this relationship is heavily influenced by other factors, which may in fact be on the "causal pathway" between increasing age and reduced PHI. For example, older age generally results in lower income and this lower income may then influence the probability of PHI uptake. It is interesting to note that once sex, remoteness, income, education, aboriginality, relationship status and country of birth were taken into account, older study participants were significantly *more *likely to have PHI than younger ones. Considered together, these findings suggest that the lower absolute PHI coverage in older participants may be explained primarily by factors accompanying ageing (e.g. low income, being single) and, at the same time, coverage is higher than expected once these factors are taken into account. This means that other influences, such as rebates, lifetime cover incentives, differences in risk perception and health consciousness may be maintaining PHI in older age.

From a policy perspective, it is the absolute proportion of those with PHI that is most important. The lower coverage with increasing age, coupled with the finding that PHI is higher in those in better health and hence less need for health care, suggests that the potential contribution of PHI to dealing with the increased morbidity associated with population ageing may be limited. Moreover, PHI does not specifically cover aged care provided by residential or community care, and specialised geriatric services are almost exclusively based in public health care facilities.

This study has a number of strengths. It provides a unique combination of large numbers and comprehensive questionnaire data, allowing investigation of the relationship between PHI and a very wide range of variables with a great deal of power and with adjustment for multiple factors. A number of factors investigated here are being reported on for the first time. The cross-sectional nature of the study means it is not possible in many cases to know whether or not the purchase of PHI came before or after the exposure in question and in these cases one cannot attribute a causal relationship to the acquisition of PHI. However, most risk factors are likely to pre-date or not be influenced substantively by PHI purchasing decisions. The self-reported nature of the variables should be considered when interpreting the findings. We have adjusted for multiple potential confounding factors, but it remains possible that other unmeasured factors influence PHI uptake.

The 45 and Up Study is a large scale cohort study and is designed to provide valid comparisons of the characteristics of groups within the cohort (e.g. relative risks of certain outcomes in exposed and unexposed individuals), rather than prevalence estimates that are representative of the general population. The absolute age-specific percentage of individuals reporting PHI observed here was higher than that reported elsewhere. Fund membership data from the Private Health Insurance Administration Council indicate that in 2007, around 51% of NSW residents aged 45 years and over had private health insurance [12,13]. In the most recent Australian National Health Survey (2004–5), 61% of respondents aged 45–54, 61% of those aged 55–64 and 51% of those aged 65–74 reported having PHI [3,14], compared to corresponding figures of 67%, 69% and 61% in the data presented here. The high rate of PHI coverage among 45 and Up Study participants is likely to reflect the well recognised "healthy cohort effect"; it does not detract from the validity of internal comparisons of relative risks of PHI in different groups [4,15].

The pattern of variation in PHI seen here by age, income, education, urban/rural residence, marital status, paid work status and country of birth is consistent with analyses of data from the Australian National Health Survey from 1989–2005 [3,16-20]. We found that PHI coverage among Indigenous people, after adjusting for other sociodemographic factors, was only half that of the non-Indigenous population. The only previous information about PHI in Indigenous people comes from a study of payment classification status for hospital episodes in Western Australia; this reported that the likelihood of Indigenous individuals using PHI for hospitalisation was negligible [21].

The strong observed relationship between PHI and socioeconomic status is not surprising, since PHI is relatively costly and there are incentives for those with higher incomes to purchase PHI. Furthermore, the main reason given for not having PHI among uninsured individuals in the 2001 National Health Survey was being unable to afford it [3]. The independent relationships of education, region of residence, marital status, paid work and country of birth, over and above income, suggests that knowledge about health, accessibility to services, social interactions and cultural factors may all play a role in decisions about whether to purchase private health insurance.

Overall, our data suggest that people with PHI are likely to be more health conscious than uninsured individuals. Our finding of lower rates of PHI in smokers is consistent with others [3,16-22]. This association held regardless of education, income and other sociodemographic factors. People's individual attitudes towards risk are likely to influence both decisions about health behaviours, such as giving up smoking, and whether or not to take out private health insurance.

In our sample, people who drank alcohol weekly were more likely than non-drinkers to have PHI. This is in apparent contrast to crude results from the 2004–5 National Health Survey, showing lower levels of PHI in heavy drinkers aged less than 65 and no difference in PHI status according to alcohol consumption in those 65 and over [3]. It is difficult to compare these results because of the differences in adjustment. While previous research has suggested increased PHI in overweight individuals compared to those of healthy weight [18], our finding of significantly reduced PHI in obese individuals, after accounting for sociodemographic factors, has not to our knowledge been shown before. Similarly, our findings of higher levels of PHI in people with higher levels of physical activity and fruit and vegetable consumption do not appear to have been reported elsewhere.

The relationship between health status and PHI is complex. We found lower levels of PHI among those reporting stroke or diabetes, with worse self-rated health, with higher levels of psychological distress and with reduced functional capacity. Crude Australian National Health Survey data from 2004–5 show decreased PHI among people reporting specific long term health conditions, including ischaemic heart disease, diabetes, arthritis and mental and behavioural problems, and among people with higher levels of psychological distress [3]. Another study has reported that PHI coverage is lower in people with worse self-rated health [18].

PHI users were more likely than non-users to report non-melanoma skin and prostate cancers, after adjusting for socioeconomic and demographic factors. We are not aware of any previous publications on these relationships. These cancers are increased among those undergoing screening, so they may be related to the general health consciousness and screening behaviour of insured individuals. They are related to higher socioeconomic status [23,24], which may not have been fully accounted for in the adjustment process. The long term nature of these conditions means it is also possible that PHI may have been purchased subsequent to diagnosis.

Overall, the evidence suggests that those with disabling illness are less likely to have PHI. However, many less disabling screening-related conditions, such as hypertension and hypercholesterolaemia, are very common and it is therefore possible for PHI possession to be related to an increasing number of health conditions, at the same time as being reduced among people with certain conditions and poorer self-rated health.

The finding of higher levels of PHI among those reporting past operations for hip and knee replacement, skin cancer removal, prostatectomy and repair of uterine prolapse has not been reported previously, but are likely to relate to better accessibility to elective surgery among those with PHI. This is consistent with National Health Survey data showing higher PHI among those reporting hospitalisation within the previous 12 months [16]. The data regarding family history are difficult to interpret as many familial diagnoses (e.g. dementia, hip fracture, osteoporosis) are predominantly markers of parental longevity. The data suggest possible clustering of not having PHI, indicated by reduced insurance among those with a family history of diabetes and those with a family history of lung cancer (i.e. smoking).

## Conclusion

In summary, our findings demonstrate that, compared to the rest of the study population, those with PHI are richer, better educated, more health conscious, in better health and more likely to use certain more discretionary health services. These findings have important implications for health services planning in that they demonstrate generally higher PHI uptake among those in better health and hence less need for health care. Differential uptake of PHI will impact on the distribution of health care costs among the private and public sectors, and how this changes over time. It is likely to influence individual trajectories of health care use as people age and–ultimately–their health outcomes. These issues will be further explored in subsequent analyses using the 45 and Up Study.

## Competing interests

The authors declare that they have no competing interests.

## Authors' contributions

EB participated in the design of the study, oversaw data analysis and drafted the manuscript. LJ participated in the design of the study, oversaw data analysis and helped to draft the manuscript. SL participated in the design of the study, performed the statistical analysis and helped to draft the manuscript. KR participated in the design of the study and assisted with the statistical analysis. All authors read and approved the final manuscript.
