# Health, ageing and private health insurance: baseline results from the 45 and Up Study cohort

**DOI:** 10.1186/1743-8462-6-17

**Published:** 2009-07-28

**Authors:** Emily Banks, Louisa Jorm, Sanja Lujic, Kris Rogers

**Affiliations:** 1 National Centre for Epidemiology and Population Health, Australian National University, ACT 0200, Australia; 2 The Sax Institute, P.O. Box 123, Broadway NSW 2007, Australia; 3 School of Medicine, University of Western Sydney, Campbelltown Campus, Locked Bag 1797, Penrith South DC NSW 1797, Australia

## Abstract

Correction to Banks E, Jorm L, Lujic S, Rogers K. Health, ageing and private health insurance: baseline results from the 45 and Up Study cohort. ANZ Health Policy 2009; 6: 16.

## Correction

In our paper on health, ageing and private health insurance [1] the second sentence of the second paragraph of the Statistical Methods should read: "All statistical tests were two-sided, using a significance level of p < 0.05." Hence, all of the confidence intervals shown in tables 1, 2 and 3 are 95% confidence intervals.
